# Data of multilayer mesh NoC performance analysis for throughput and delay over FTP and CBR applications

**DOI:** 10.1016/j.dib.2022.108196

**Published:** 2022-04-27

**Authors:** Charanarur Panem, Udaysing V. Rane, Rajendra S. Gad

**Affiliations:** Altera SOC Lab, SPAS, Electronics, Goa University, Goa, India

## Abstract

This work demonstrates the Network on Chip(NoC) performance evaluation parameters matrix-like throughput and latency of multilayer NoC Mesh topology for random break-in links(0,5,10,15,20%) over constant bit rate (CBR) and file transfer protocol (FTP) applications. The said study is further extended for 3 dimensional (3D) multilayer NoC Mesh topology with normal channels and without virtual channels over CBR and FTP traffic. These virtual channels are introduced over 2 layer 3D NoC model over-center sphere links, mid sphere links, peripheral sphere links, and all combined channels over various sphere links. The data for the 3D NoC model proposed was processed using Network Simulator-3(NS-3) software, while the throughputs and delay were processed with NS-2 Wireless Trace Analyzer.

## Specifications Table

**Table:** 

Subject area	Microelectronics Engineering
Specific subject area	Network on Chip(NoC)
Type of data	Numeric data tables
How data was acquired	Throughput and Delay for FTP and CBR applications over the proposed model were computed using Network Simulator-2.5(NS-2.5), NS-2 Wireless Trace Analyzer
Data format	Raw Integer and Real numbers
Description of data collection	The observed apparent Throughput data and Delay data set were processed for FTP and CBR applications with varying Packet Size. Throughput and Delay performance matrix generated for FTP and CBR for random break-in links, with normal and virtual channels over multilayers NoC
Data source location	ALTERA SOC Lab, Electronics, School of Physical and Applied Sciences, Goa University, Goa, India.
Data accessibility	All the data are with the article and https://github.com/panemcharan/Data-of-Multilayer-Mesh-NoC-Performance-Analysis-for-Throughput-and-Delay-over-FTP-and-CBR-applicati
Related research article	**For a co-submission:-** Panem, C., Gad, R. S., & Kaushik, B. K. (2021). Vertical traversal approach towards TSVs optimization over multilayer network on chip (NoC). Microelectronics Journal, 116, 105,231. https://doi.org/10.1016/j.mejo.2021.105231

## Value of the Data

• The dataset can be used for modeling throughput and delay over FTP and CBR applications to evaluate the performance of the multilayer NoC.
• The dataset can be used for the Quality of Service of Network on Chip, with the parameters like a break in nodes and links in a mesh topology.
• The dataset can be used to customize the density of the Through Silicon Via(TSV) Keep-out Zone.
• The dataset can be used to develop data communication protocols in a multilayer NoC environment for the development of an advanced computing system.
• The data can be compared with a similar performance matrix obtained by various researchers in multilayer NoC Mesh topologies.

## 1 Data Description

The present tables contain 3D 2-layer NoC Mesh topology for random break-in links (0,5,10,15,20%) over nodes of the NoC network.

Performance matrix in terms of throughput and delay over multilayer NoC Mesh topology with normal channels and with virtual channels.

The Table 1 represents the various parameters which are customized for 3D NoC model over FTP and CBR traffic applications. The major parameters are type of topology, communication element, transmission protocol, routing scheme, routing protocol, queue mechanism, simulation time and numbers of nodes in Mesh topology. These parameters are selected to generate three major scenarious described in Tables 2–7. These major scenarios are consists of throughput and delay performance for FTP and CBR applications, The Table 2 describes the throughput versus packet size for 2 layer NoC Mesh topology for random break-in links(0%,5%,10%,15%, and 20%) over FTP and CBR traffic applications, and Table 3 presents the delay versus packet size for 2 layer NoC Mesh topology for random break-in links(0%,5%,10%,15%, and 20%) over FTP and CBR traffic application. The said scenario is extended further with 2 to 8 multilayer NoC Mesh topology with a virtual channel and with normal channels again over FTP and CBR traffic with the variation of throughput versus packet size presented in Table 4, and Table 5 shows the data delay versus packet size. Later the study is extended for the 18 × 18 nodes with 2 layer NoC model having one vertical channel at center sphere link, 4 vertical channel at mid sphere links, 4 vertical channel at periphery sphere links, and all channel combines, over FTP traffic throughput v/s packet size describes the data in Table 6 and the Table 7 represents traffic delay v/s packet size . The present data describes the data in three case studies. Case study I describe the data of 3D 2-layer NoC Mesh topology for random break-in links, case study II presents data with normal channels and with virtual channels, and case study III explain the data vertical channel at the center sphere link, four vertical channel at mid sphere links, four vertical channel at periphery sphere links.

## 2 Experimental Design, Materials, and Methods

We consider some variants of 3D Mesh topologies, i.e., 2,4,8 layer; in this data representation. The multilayer variants are considered to demonstrate the TSV's link effectiveness between the layers. The example is a 2-dimensional mesh topology; each router is routed to four nearby routers and a computational resource. The number of routers is equal to the number of computational resources. The computational resources and the routers are connected through communication channels. Each channel has two unidirectional links between two routers or between a router and a computational resource. We use the deterministic XY routing algorithm for routing over this topology. The vertical channels, i.e., up and down, establish a connection between the 2-dimensional layers. The performance parameter, i.e., a throughput, is the total number of received packets by the destinations per unit time. When one or more packets do not reach their destination due to the contention over a network link or lack of buffer space, the error is introduced over the network, and hence there is packet loss. A common tool used to simulate small and large area networks is NS-3(https://www.nsnam.org/). Since there are similarities between NoCs and networks, NS-3 is preferred by researchers to understand behaviors at a higher abstraction level of design. There are various topologies and protocols support, and even customized protocols can be incorporated. The parameters for routers and links can easily be scaled to simulate the real situation on a chip shown in Table 1. With this understanding, we have simulated a 4 × 4 and 18 × 18 nodes multilayer mesh NoC for performance analysis for packet delivery.

Table 1. Various parameters 3D NoC over FTP and CBR traffic applications.

Case Study 1: Throughput,Dealy v/s Packet size for 2 layer NoC Mesh topology for random break in links(0,5,10,15,20%) over FTP and CBR traffic application.

Table 2. Throughput v/s Packet size for 2 layer NoC Mesh topology for random break-in links(0,5,10,15,20%) over FTP and CBR traffic application.

Table 3. Delay v/s Packet size for 2 layer NoC Mesh topology for random break-in links(0,5,10,15,20%) over FTP and CBR traffic application.

Case Study 2: Throughput, Delay v/s Packet size for 2 to 8 multilayer NoC Mesh topology with a virtual channel and with normal channels over FTP and CBR traffic.

Table 4. Throughput v/s Packet size for 2 to 8 multilayer NoC Mesh topology with a virtual channel and with normal channels over FTP and CBR traffic.

Table 5. Delay v/s Packet size for 2 to 8 multilayer NoC Mesh topology with a virtual channel and with normal channels over FTP and CBR traffic.

Case Study 3: Throughput, Delay v/s Packet size for 18 × 18 nodes 2 layer NoC model having one vertical channel at center sphere link, 4 vertical channel at mid sphere links, 4 vertical channel at periphery sphere links, and all channel combines over FTP traffic.

Table 6. Throughput v/s Packet size for 18 × 18 nodes 2 layer NoC model having one vertical channel at center sphere link, 4 vertical channel at mid sphere links, 4 vertical channel at periphery sphere links, and all channel combines over FTP traffic.

Table 7. Delay v/s Packet size for 18 × 18 nodes 2 layer NoC model having one vertical channel at center sphere link, 4 vertical channel at mid sphere links, 4 vertical channel at periphery sphere links, and all channel combines over FTP traffic.

Observations: As more horizontal links are disconnected randomly, the shortest path from source to a destination increases due to more numbers of hops, and the throughput decreases further below 600 KBps for FTP and CBR. Therefore, it is a reasonable conclusion that FTP and CBR are capable of exploiting the bandwidth at reasonable packet size in 2 layer multilayer NoC shown in Table 2. Consider specifically the 20% break-in links case for FTP and CBR traffic 25 ms is the highest delay value obtained for 4000 KB, while 21 ms and 17 ms is the value of delay for 15% and 10% break-in links, respectively. Above 4000 KB packets size, the network follows square law. This is due to the bottleneck of the resources (like buffer size, link bandwidth, etc.) assigned to the routers [1].

3-D NoC network latency performs linearly as the function of packet size delay. Also, in the multilayer NoC, the latency factor with virtual channels is always marginally higher than with normal channels due to the additional resource of virtual channels. The result for throughput and latency are in agreement with [2,3] effective utilization of bandwidth resources.

Evaluation of 2 layers 18 × 18 nodes mesh NoC topology over the placement of virtual channels (i.e., vertical channels connecting layers) from various positions (i.e., center, mid-sphere, and periphery over the sphere) is studied. The study is performed in independent and combination channel modes for FTP traffic. The 3-D routers placed over spheres from the center, the performance is lowest for central sphere router as compared to peripheral sphere router shown in Tables 6 and 7 [4].

The present study consists of 3D NoC Throughput and Delay for FTP and CBR applications; the data set for Throughput and delay measured with network simulator 2.5(NS-2.5) using network trace analyzer. Throughput and latency of 3D 2-layer NoC Mesh topology for random break-in links (0,5,10,15,20%) over CBR and FTP traffic data set for throughput and delay are shown in Tables 2 and 3.

3D multilayer NoC Mesh topology with normal channel and with virtual channels over CBR and FTP traffic data set for throughput and delay shown in Tables 4 and 5. The 2 layer 3D NoC model has one vertical channel at the center sphere link, 4 vertical channels at mid sphere links, 4 vertical channels at periphery sphere links, and all channels combined over FTP traffic data set for throughput and delay shown in Tables 6 and 7. The combined 3D NoC throughput and delay over FTP, CBR applications with different case studies have been used to study the performance evolution of NoC and quality of service [5].

## Ethics Statements

It does not apply to this dataset.

## CRediT authorship contribution statement

**Charanarur Panem:** Conceptualization, Methodology, Software. **Udaysing V. Rane:** Writing – review & editing. **Rajendra S. Gad:** Supervision.

## Declaration of Competing Interest

The authors declare that they have no known competing for financial interests or personal relationships which have, or could be perceived to have, influenced the work reported in this article.

## Data Availability

Data-of-Multilayer-Mesh-NoC-Performance-Analysis-for-Throughput-and-Delay-over-FTP-and-CBR-applicati (Original data) (https://github.com/).
